# Safety and efficacy of intrathecal methotrexate in severe anti-NMDAR encephalitis: a single-institution retrospective study

**DOI:** 10.3389/fimmu.2026.1936870

**Published:** 2026-10-26

**Authors:** Minghui Tuo, Jiabin Fang, Yuanteng Fan, Yan Xu

**Affiliations:** 1 Department of Neurology, Wuhan Zhongnan Hospital of Wuhan University, Wuhan, Hubei, China; 2 Hubei Provincial Clinical Research Center for Dementia and Cognitive Impairment, Wuhan, China; 3 Department of Neurology, Wuhan Fourth Hospital, Wuhan, Hubei, China

**Keywords:** anti-NMDAR encephalitis, autoimmune encephalitis, immunotherapy, intrathecal injection, methotrexate

## Abstract

**Background:**

Anti-N-methyl-D-aspartate receptor (anti-NMDAR) encephalitis is one of the most common forms of autoimmune encephalitis (AE). Most cases can achieve clinical relief with first- and second-line immunotherapies, while approximately 25% are resistant to these immunotherapies and require further immunomodulatory therapy. Intrathecal methotrexate (IT-MTX) is one of the escalation treatments. This study aimed to analyze the safety and efficacy of IT-MTX in severe cases of anti-NMDAR encephalitis.

**Methods:**

A retrospective observational analysis was performed on 10 patients with severe anti-NMDAR encephalitis who received IT-MTX weekly for 4 consecutive weeks in our institution between 2021 and 2024. We reviewed patients’ baseline clinical characteristics, cerebrospinal fluid antibody titers, modified Rankin Scale (mRS) scores, and Clinical Assessment Scale in Autoimmune Encephalitis (CASE) scores before and after IT-MTX. The entire follow-up observation lasted for 12 months.

**Results:**

Nine of 10 patients experienced significant clinical relief at the 12-month follow-up after IT-MTX, with a significant decrease in CASE scores (6–24 to 0-2) and mRS scores (4–5 to 0-1) in survivors, and six of 10 patients had a parallel decrease in antibody titers at the 12-month follow-up. No IT-MTX-related neurotoxicity or recurrences were observed during the 1-year follow-up. One patient who did not complete the standard IT-MTX treatment died from a severe systemic infection, which was considered unrelated to IT-MTX. When patients receiving IT-MTX within 2 weeks were compared with those receiving IT-MTX >2 weeks after conventional immunotherapy, reductions in CASE and mRS scores were larger in the early-treatment group (p < 0.05).

**Conclusion:**

Our findings suggest that IT-MTX may be an effective and relatively safe escalation option for severe anti-NMDAR encephalitis. Early and aggressive application of IT-MTX may improve the prognosis. Further large prospective studies are needed to better illustrate the safety, efficacy, and optimal timing of IT-MTX in severe anti-NMDAR encephalitis.

## 1 Introduction

Autoimmune encephalitis (AE) is a group of immune-mediated inflammatory disorders of the brain parenchyma with clinical heterogeneity (1). Anti-N-methyl-D-aspartate receptor (anti-NMDAR) encephalitis, one of the most common forms of AE, was first described in 2007 (2). It is associated with cerebrospinal fluid (CSF) IgG antibodies against the GluN1 subunit of the NMDA receptor. Typically, it has an acute or subacute onset and presents diverse clinical manifestations, including psychiatric symptoms, cognitive impairment, seizures, motor dysfunction, and autonomic instability. It occurs more frequently in young women and is associated with underlying tumors, most commonly ovarian teratomas (3).

First-line immunotherapies for AE include glucocorticoids, intravenous immunoglobulin (IVIG), plasma exchange (PLEX), and immunoadsorption alone or in combination. If there is no improvement within 2 weeks after first-line therapy, second-line agents should be initiated, including rituximab or cyclophosphamide (4, 5). Most patients experience clinical relief following first- and second-line therapies, but a minority fail to respond to these therapies. This leads to prolonged Intensive Care Unit stays and severe disability (6). The common escalation therapies include intrathecal methotrexate (IT-MTX), tocilizumab, bortezomib, low-dose IL-2, etc. In severe cases, how to choose the treatment strategy remains controversial (7).

MTX is an anti-folate metabolite used in chemotherapy and immunosuppression in autoimmune diseases, and its intrathecal administration leads to higher local drug concentrations and minimal systemic toxicity (6). According to previous case reports (8), most patients with anti-NMDAR encephalitis showed significant improvement and a decrease in CSF antibody titers after IT-MTX, with no serious side effects observed. However, the evidence for the efficacy of IT-MTX in patients with severe anti-NMDAR encephalitis is mainly based on case reports or very small case series.

This study aimed to retrospectively summarize and evaluate the safety and efficacy of IT-MTX in treating severe anti-NMDAR encephalitis at our institution.

## 2 Patients and methods

### 2.1 Participants

We included 10 patients who met the diagnostic criteria for anti-NMDAR encephalitis (9) and received IT-MTX in the Department of Neurology, Zhongnan Hospital of Wuhan University, from 2021 to 2024. The included patients had completed at least 12 months of follow-up after discharge, except for one patient who died. This study was approved by the Medical Ethics Committee of Zhongnan Hospital of Wuhan University, and the requirement for informed consent was waived (2025378K). The diagnostic criteria for anti-NMDAR encephalitis were as follows: (1) rapid onset (less than 3 months) of one or more of the six major groups of symptoms, including abnormal (psychiatric) behavior or cognitive dysfunction, speech dysfunction (pressured speech, verbal reduction, and mutism), seizures, movement disorders (dyskinesias or rigidity/abnormal postures), decreased level of consciousness, and autonomic dysfunction or central hypoventilation; the presence of NMDAR antibody; exclusion of other diseases. Severe anti-NMDAR encephalitis (10) was defined as anti-NMDAR encephalitis fulfilling one or more of the following criteria: (1) Impaired consciousness (Glasgow Coma Scale score ≤ 12); (2) Status epilepticus; (3) Dystonic status (Dystonia Severity Action Plan grade 4–5); (4) Psychiatric symptoms requiring management in the Neurological Critical Care Unit; (5) Severe autonomic dysfunction resulting in hemodynamic instability; (6) Central hypoventilation syndrome.

### 2.2 Methods

Clinical data of patients were collected, including demographic data, clinical manifestations, laboratory examinations, imaging findings, electroencephalogram (EEG), and follow-up data. All patients underwent computed tomography (CT), magnetic resonance imaging (MRI), or ultrasonography of the abdomen and pelvis for malignancy screening. Anti-NMDAR antibodies were evaluated using a cell-based assay (CBA) (Guangzhou KingMed Diagnostics Group Co., Ltd., Guangzhou, Guangdong, China) in the CSF of each patient. The initial dilution titers were 1:1 for CSF. For CSF antibody titers, 1:1 was defined as weakly positive, 1:3.2 to 1:10 as positive, and titers of 1:32 and above as strongly positive. All serial paired CSF samples collected during follow-up were tested using the identical CBA platform to avoid inter-assay variability. We tested the antibody titers and recorded the neurological status using the modified Rankin Scale (mRS) and the Clinical Assessment Scale in Autoimmune Encephalitis (CASE) scores (11) at regular intervals (before IT-MTX, after the last IT-MTX, and at 6 and 12 months post-treatment).

### 2.3 Treatment

The first-line immunotherapy included high-dose methylprednisolone (1000 mg/d, reduced by half every 3 days), IVIG (5 days, 0.4 g/kg/d), and/or PLEX; second-line treatment included rituximab or ofatumumab (20 mg). IT-MTX was administered after failure of first-line or second-line treatments. Seven of the 10 participants received weekly intrathecal injections of 10 mg of MTX and 5 mg of dexamethasone for 4 weeks (12). Case 2 died after the second IT-MTX injection because of severe systemic complications; Case 5 completed only three injections due to technical difficulties in intrathecal administration caused by severe obesity, and Case 8 completely recovered after the third injection. All patients continued to receive oral 1mg/kg/day corticosteroids (tapered by 5 mg every 2 weeks), mycophenolate mofetil, ofatumumab, antiepileptic drugs, or psychiatric therapy during and after IT-MTX therapy according to patients’ conditions.

### 2.4 Follow-up

After initiation of intrathecal therapy, patients were followed up for at least 12 months either in the outpatient department or during hospital admission. All patients underwent detailed neurological examinations, electroencephalography (EEG), and brain MRI at treatment initiation and during subsequent follow-up to detect any subclinical methotrexate-induced toxicity, including chemical meningitis, delayed leukoencephalopathy, and transverse myelopathy.

### 2.5 Statistical analysis

Statistical analyses were performed using GraphPad Prism 6. Analysis of continuous data between subgroups was performed using the unpaired Student’s t-test. p < 0.05 indicated statistical significance.

## 3 Results

### 3.1 Baseline clinical characteristics

From January 2021 to December 2024, a total of 10 patients were enrolled in this study (**Table 1**). The median age was 22.6 years (14–31 years); three patients were male and seven were female. At the onset of the disease, six patients manifested symptoms resembling a viral infection, including headaches and fevers. Two patients presented with epileptic symptoms, while five patients displayed psychiatric and behavioral abnormalities. As the disease progressed, symptoms of cognitive impairment, such as memory loss and inattentiveness, emerged in seven patients. Epileptic symptoms were experienced by eight patients, and nine patients exhibited psychiatric and behavioral aberrations, ranging from visual hallucinations, incoherent speech, and mania, to irritability. Moreover, seven patients suffered from varying degrees of consciousness impairment, presenting as somnolence or coma.

**Table 1:** Clinical presentation of 10 patients with anti-NMDAR encephalitis.

Items	Case 1	Case 2	Case 3	Case 4	Case 5	Case 6	Case 7	Case 8	Case 9	Case 10
Gender	Male	Female	Male	Female	Male	Female	Female	Female	Female	Female
Age (years)	14	20	17	27	17	24	31	29	23	24
Initial symptoms	Headache, fever, psychosis	Fever	Fever, headache	Headache, fever	Disturbance of consciousness, seizures	Headache, psychosis	Headache, limb weakness	Psychosis	Psychosis	Seizures, psychosis
Primary symptoms	Seizures, autonomic dysfunction, cognitive impairment, coma	Fever, seizures, memory impairment, psychosis, coma	Fever, seizures, memory impairment, visual hallucinations, drowsiness	Seizures, memory impairment, incoherent speech	Seizures, cognitive impairment, agitation, coma	Headache, cognitive impairment, psychosis	Seizures, incoherent speech, slowed responses, coma	Psychosis, confusion	Seizures, psychosis	Seizures, psychosis, cognitive impairment, somnolence
Time from onset to diagnosis (days)	14	11	9	19	10	8	10	26	3	25
CSF antibody titers										
Initial	1:1	1:32	1:3.2	1:100	1:320	1:32	1:32	1:32	1:100	1:100
Before IT-MTX	1:100	1:100	1:10	1:1	1:10	1:32	1:100	1:32	1:32	1:100
After last IT-MTX	1:10	1:10	1:10	1:1	1:10	1:32	1:32	1:1	1:10	1:100
6 months after IT-MTX	1:1	Died	1:1	NE	1:1	1:32	1:32	Negative	1:10	Negative
EEG	Mild abnormality	Severe epileptiform discharges	Severe abnormality	Moderate epileptiform discharges	Mild abnormality	Severe epileptiform discharges	Moderate abnormality	Mild abnormality	Moderate abnormality	Severe epileptiform discharges
Brain MRI	Abnormal	Normal	Abnormal	Abnormal	Normal	Normal	Abnormal	Abnormal	Normal	Abnormal
Tumor resection	None	Teratoma	None	None	None	Teratoma	Teratoma	None	None	None
Complications and concomitant conditions	Infection, respiratory failure, malnutrition	Lower extremity venous thrombosis, infection, severe anemia	Pulmonary infection	None	Urinary tract infection, pulmonary infection, malnutrition	Pulmonary infection, hypoalbuminemia	Pulmonary infection, fungal infection	Pulmonary infection	Pulmonary infection, hypoalbuminemia	Pulmonary infection, stress ulcer, respiratory and circulatory failure
First-line immunotherapy	IVMP + IVIG + PLEX	IVIG + IVMP + PLEX	IVMP + IVIG	IVMP + PLEX	IVMP + IVIG + PLEX	IVMP + IVIG + PLEX	IVMP + IVIG	IVMP + IVIG	IVMP + IVIG	IVMP + IVIG
Second-line immunotherapy	rituximab	rituximab + efgartigimod	None	None	rituximab	None	rituximab	ofatumumab	None	None
Time between IT-MTX and previous immunotherapy (days)	52	37	14	54	11	14	10	10	5	3
IT-MTX regimen	Weekly for 4 weeks	2 doses	Weekly for 4 weeks	Weekly for 4 weeks	3 doses	Weekly for 4 weeks	Weekly for 4 weeks	Weekly for 3 weeks	Weekly for 4 weeks	Weekly for 4 weeks
CASE score										
Before IT-MTX	24	27	17	10	16	18	21	6	11	24
After last IT-MTX	23	27	7	6	8	4	4	0	1	4
6 months after IT-MTX	9	Died	0	3	2	1	1	0	1	3
12 months after IT-MTX	2	Died	0	1	1	0	0	0	0	1
mRS score										
Before IT-MTX	5	5	5	3	5	5	5	4	4	5
After last IT-MTX	5	5	3	2	3	2	3	0	1	2
6 months after IT-MTX	2	6	0	1	1	1	1	0	1	1
12 months after IT-MTX	1	6	0	1	1	0	0	0	0	1

IVIG, intravenous immunoglobulin; IVMP, intravenous methylprednisolone pulse; PLEX, plasma exchange; NE, not evaluated..

Initial EEG and brain MRI were performed in all 10 patients before immunotherapy treatment. EEG revealed mild to severe abnormalities in all patients, with epileptiform discharges detected in four cases. Six patients demonstrated high signal intensity on brain MRI T2 or T2-FLAIR sequences, primarily localized in the periventricular white matter, hippocampus, and temporal-parietal-occipital regions.

Three ovarian teratomas were removed immediately after being detected. One patient was found to have a teratoma at the 17-month follow-up. The histopathology revealed mature cystic teratomas, and in two of these teratomas, mature brain tissue was observed.

### 3.2 NMDAR antibody titers in CSF

All patients exhibited varying degrees of increase in NMDAR antibody titers in their CSF. Following first-line and/or second-line immunotherapy, antibody titers decreased in three patients, remained unchanged in three patients, and increased in four patients. Following IT-MTX treatment, antibody titers decreased significantly in five patients, and in another three patients, antibody titers decreased 6 months later. One patient showed decreased titers at the 4-month post-discharge follow-up but exhibited increased titers at the 8-month follow-up. One patient had unchanged titers at the last IT-MTX treatment but subsequently refused to undergo lumbar puncture to test the CSF antibody titers (**Figure 1**).

**Figure 1:**
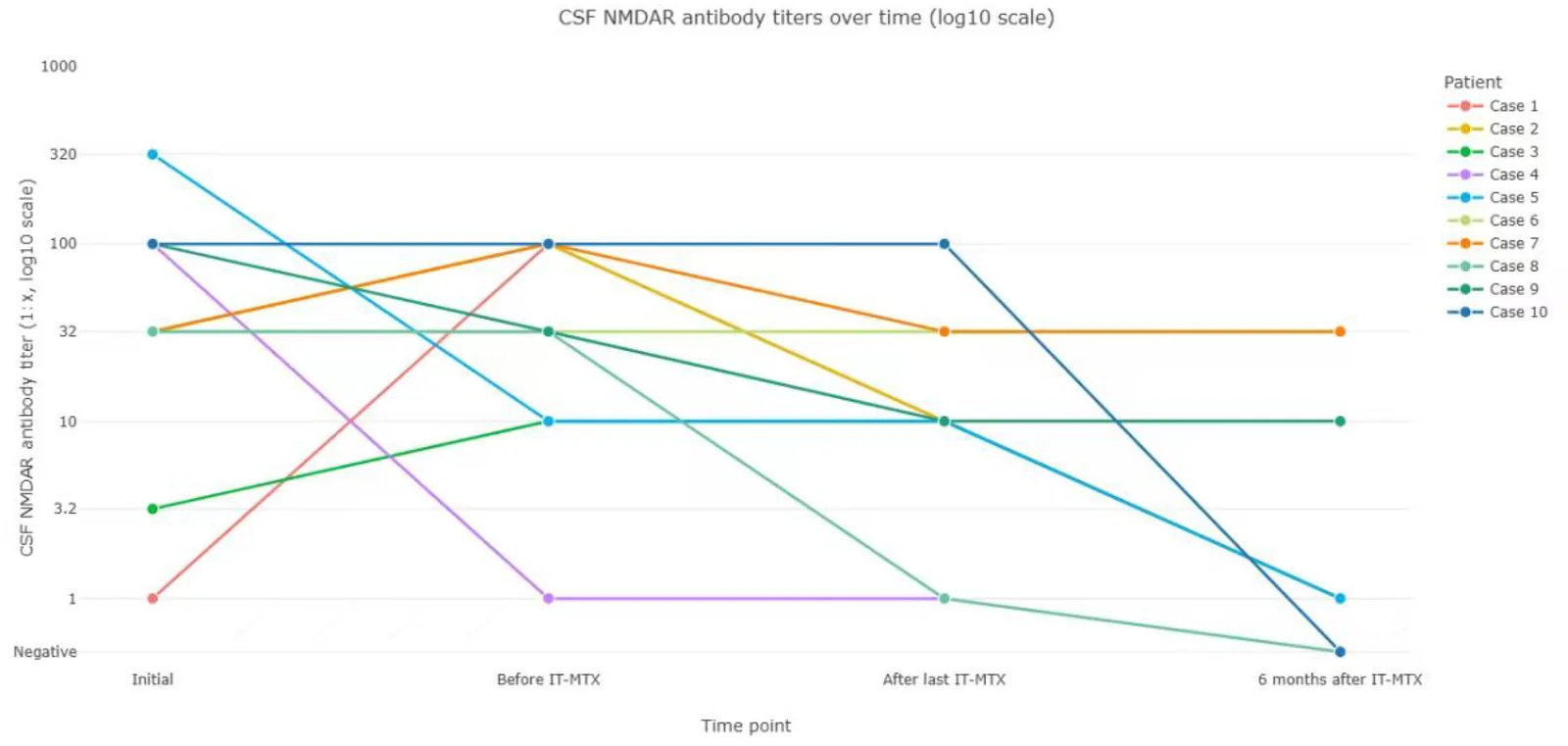
Changes in CSF antibody titers in 10 patients.

### 3.3 Treatment and follow-up

The 10 patients were diagnosed within a range of 3 to 26 days. After diagnosis, nine patients immediately received intravenous corticosteroids in combination with IVIG treatment. Among them, four patients underwent PLEX. One patient was treated with only corticosteroid therapy combined with PLEX (**Table 1**).

Five patients received second-line drugs (rituximab or ofatumumab) after failure of first-line immunotherapy. The other five patients received IT-MTX and dexamethasone directly after first-line treatment due to severe pneumonia. All nine patients continued long-term maintenance immunotherapy, including low-dose glucocorticoids and mycophenolate mofetil or ofatumumab after discharge.

After the last IT-MTX treatment, two patients emerged from coma and the other six patients were able to follow simple commands and had appropriate interactions with others (mRS of 0-3, CASE of 0-8). Among these, one patient achieved complete recovery. However, one patient remained in a coma, and another died from a severe systemic infection unrelated to IT-MTX after the second intrathecal injection. At the 6-month follow-up, the other nine patients achieved an mRS score of 0-2. The CASE score decreased from a median of 17 before intrathecal injections to 1 (**Figure 2**), demonstrating excellent composite clinical functional improvement. At the 12-month follow-up, five patients had fully resumed their daily activities (mRS of 0, CASE of 0), and four patients had no impairment in daily activities but had mild cognitive dysfunction (mRS of 1, CASE of 1-2).

**Figure 2:**
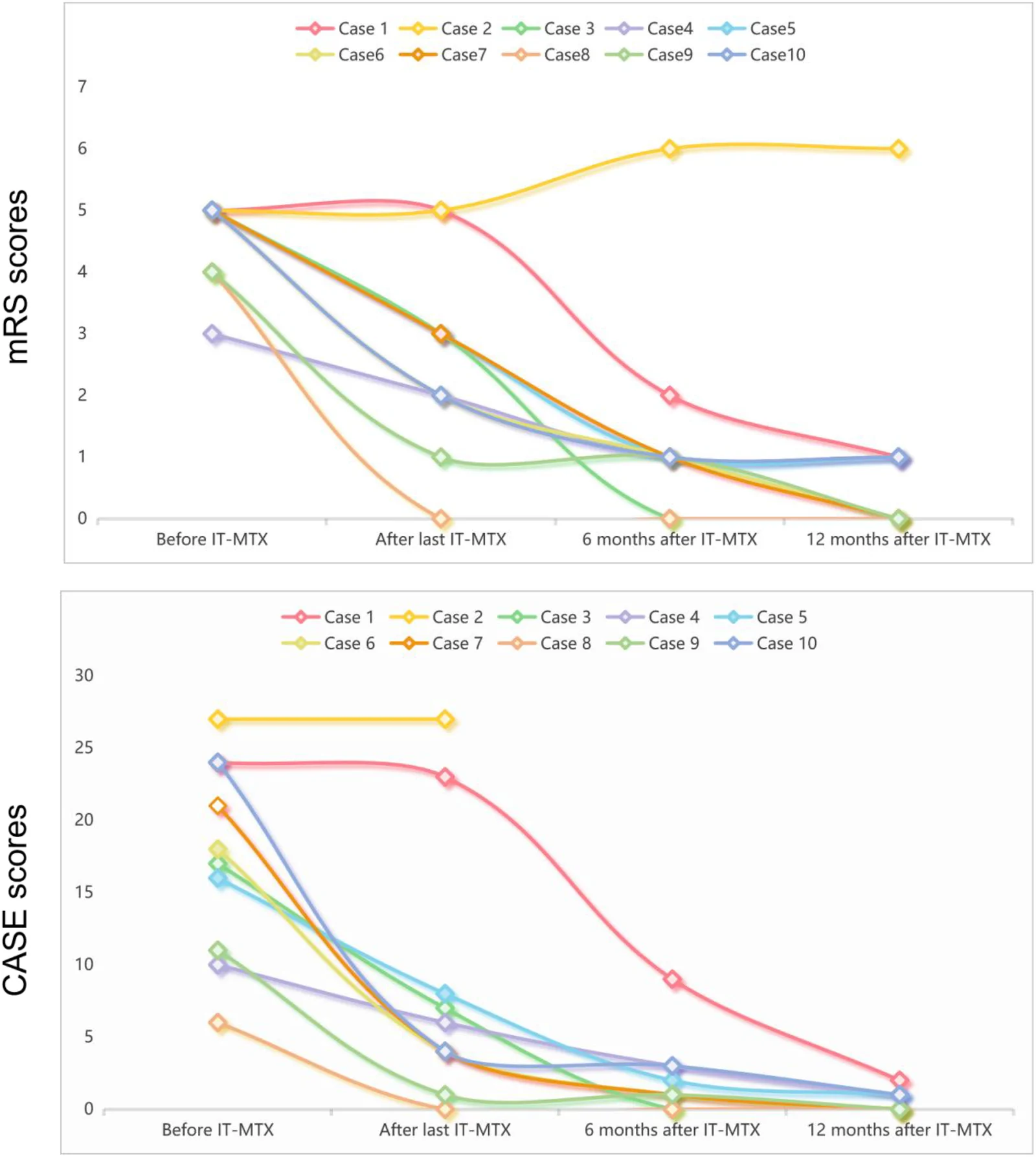
Changes in mRS and CASE scores in 10 patients with anti-NMDAR encephalitis.

To evaluate the effect of the timing of the initiation of IT-MTX, patients were stratified into two groups: those receiving sequential IT-MTX within 2 weeks (n = 7) and those receiving sequential IT-MTX beyond 2 weeks (n = 3) after first- or second-line immunotherapy. We observed greater declines in mRS scores when IT-MTX was initiated within 2 weeks after first- or second-line therapy; the mRS scores decreased significantly at 6 months and 12 months after IT-MTX (**Figure 3a**), while the CASE scores decreased immediately after the last IT-MTX treatment and at 6 and 12 months after IT-MTX (**Figure 3b**).

**Figure 3:**
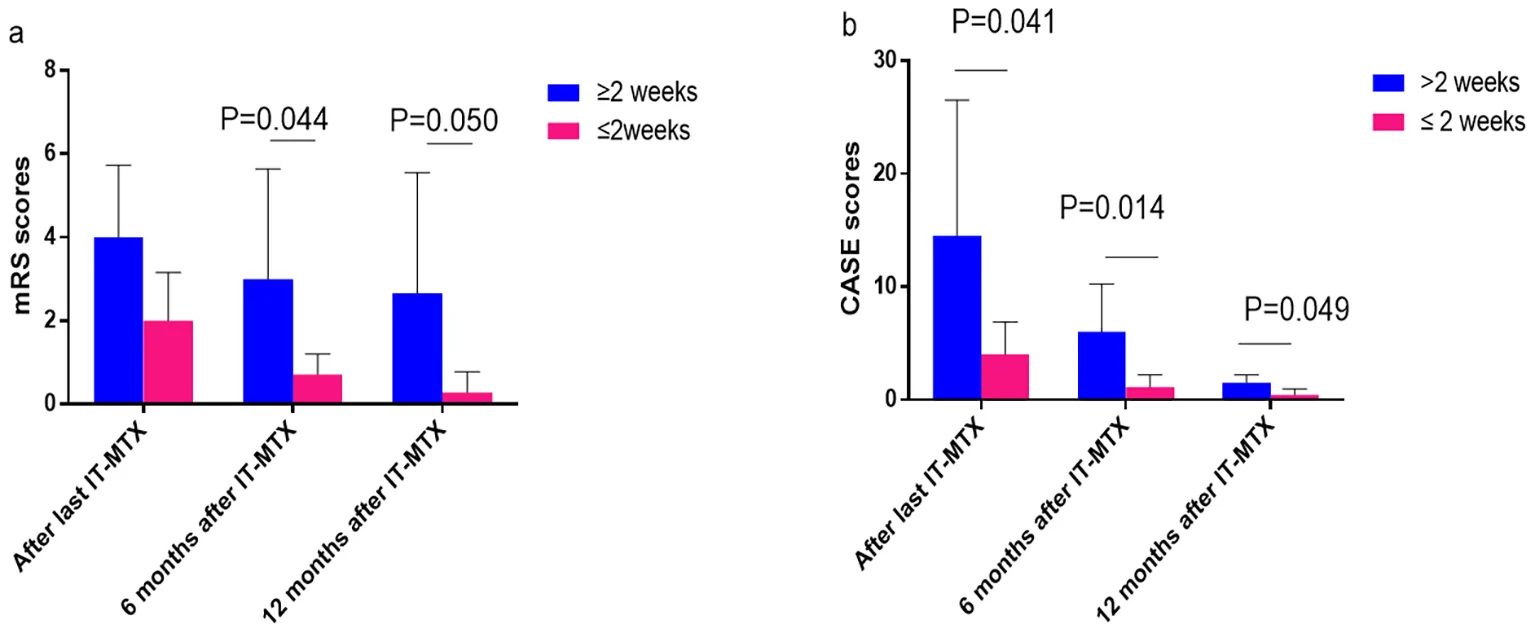
The impact of the initiation time of IT-MTX on mRS and CASE scores in patients with anti-NMDAR encephalitis. Changes in CASE and mRS scores at different IT-MTX initiation times.

## 4 Discussion

In the present study, we observed remarkable clinical improvement in anti-NMDAR encephalitis following IT-MTX, accompanied by a parallel decrease in CSF antibody titers with no adverse effects or relapses during the 12-month follow-up. Furthermore, we found that patients who received IT-MTX earlier after conventional immunotherapy (≤2 weeks) had a faster recovery and a better prognosis than those who received IT-MTX later (> 2 weeks), suggesting that intrathecal therapy should be initiated earlier after conventional immunotherapy. Although severe infections limited the application of second-line treatment in five cases, the significant clinical improvements and no adverse effects were also observed in patients who received IT-MTX following first-line treatment without second-line treatment. Therefore, IT-MTX may be an effective and safe treatment for severe anti-NMDAR encephalitis and should be used earlier after second-line therapy or after first-line therapy when second-line therapy is limited. However, because this was a small retrospective study lacking a control group and involving concurrent administration of multiple immunotherapies, more prospective studies with a larger sample size are needed to better illustrate the safety and efficacy of IT-MTX in severe anti-NMDAR encephalitis.

IT-MTX was considered one of the important escalation options for severe anti-NMDAR encephalitis (8). Intrathecal synthesis of anti-NMDAR antibodies has been firmly established in anti-NMDAR encephalitis (1, 13). The alterations in CSF antibody titers are closely correlated with the clinical prognosis. Patients with persistent symptoms often display sustained elevations in CSF antibodies (14, 15). Currently, the first- and second-line immunotherapies mainly aim to neutralize circulating antibodies and eliminate CD20^+^ B cells. However, they do not target mature long-lived plasma cells (4). Ofatumumab has been considered a novel second-line immunotherapy for severe anti-NMDAR encephalitis, effectively mediates circulating CD20^+^ B cells through complement-dependent cytotoxicity and antibody-dependent cytotoxicity (16). In our study, a patient showed no significant improvement after treatment. Various novel therapeutic targets are currently coming into use or under investigation. Daratumumab is a human monoclonal antibody targeting CD38, which is currently approved for refractory multiple myeloma. However, persisting anti-NMDAR titers after treatment indicate non-depleted long-lived plasma cells (17), with 10–17% of patients developing severe infection-related complications. For patients with severe anti-NMDAR encephalitis who are already on mechanical ventilation or have developed pulmonary infections, the risk of respiratory tract infection may be correspondingly elevated (18). Inebilizumab targets CD19^+^ B cells, including plasmablasts and some plasma cells, leading to broader and more sustained suppression of B-cell activity. It has been approved for the treatment of neuromyelitis optica spectrum disorder, and a Phase II clinical study—the ExTINGUISH trial (NCT04372615)—is currently underway to assess the efficacy and safety in anti-NMDAR encephalitis (19). The 26S proteasome inhibitor bortezomib has shown promising efficacy in severe anti-NMDAR encephalitis as it disrupts the survival of long-lived plasma cells. A prospective study failed to confirm that bortezomib treatment shortens the course of the disease, possibly due to its limited ability to cross the blood-brain barrier (BBB), and adverse reactions occurred in 37.9% of patients (20). Therefore, intrathecal treatment should be considered, as it can directly inhibit nucleic acid synthesis and cell division of intrathecally proliferating B lymphocytes and plasmablasts in CSF, thereby suppressing intrathecal anti-NMDAR antibody production, modulating immune dysregulation, and reducing the adverse effects related to systemic medication (6, 21). Although we observed reductions in antibody titers following IT-MTX treatment, antibody titer reduction cannot be solely attributed to IT-MTX. All patients received concomitant maintenance immunotherapies, and antibody fluctuations may also reflect the natural disease course.

IT-MTX for severe anti-NMDAR encephalitis has shown promising results. Nevertheless, these studies are limited by predominantly retrospective designs, small sample sizes, a lack of reported side effects, and an absence of follow-up data on complications beyond 1 year after IT-MTX (8, 12, 22–24). To date, over 20 cases of IT-MTX treatment for anti-NMDAR encephalitis have been reported, predominantly in pediatric patients, with fewer reports involving adults. IT-MTX was administered weekly for 4 weeks (12) or monthly over 6 months (23). The patients achieved a good response accompanied by a reduction in mRS and CASE scores following IT-MTX treatment without adverse reactions. Only Yang et al. (12) reported a patient who died from neurological complications 2 months later despite IT-MTX, potentially due to delayed initial treatment. Remarkable clinical improvement was observed in severe anti-NMDAR encephalitis following IT-MTX, and no recurrences or IT-MTX-related complications were observed in our study during the 12-month follow-up, except for one patient who died due to delayed teratoma resection and severe pulmonary infection. Consequently, IT-MTX represents a viable therapeutic option for patients with severe anti-NMDAR encephalitis following first-line treatment failure.

The timing and drug selection for escalated treatment in anti-NMDAR encephalitis are influenced by multiple factors, including the severity of the disease, the patient’s response to drugs, and age (23, 25). Studies suggest that intrathecal immunotherapy should be considered for children who have an inadequate response to or relapse after the first- and second-line immunotherapies and have an mRS score ≥3 (26). In this study, all patients exhibited severe clinical symptoms after first-line and/or second-line treatment, with mRS scores ≥3 and CASE scores ≥6. Waiting at least 1–2 months to assess the efficacy of second-line immunotherapy was recommended in the Chinese Expert Consensus on the Diagnosis and Treatment of AE (10). However, in patients with severe disease, it is not appropriate to suspend the next phase of treatment for such a long time to assess treatment efficacy. In our study, we compared mRS and CASE scores between patients receiving IT-MTX ≤ 2 weeks after first- and/or second-line treatment and those receiving IT-MTX > 2 weeks. We observed greater reductions in mRS and CASE scores at 6-month and 12-month follow-up among patients who received IT-MTX ≤ 2 weeks after conventional immunotherapy. This finding is consistent with a previous study by Professor Lee in South Korea in 2021 who compared the efficacy of administering rituximab and/or tocilizumab immediately after first-line immunotherapy for anti-NMDAR encephalitis. The study confirmed that patients treated with methylprednisolone, IVIG, rituximab, and tocilizumab within 1 month of onset showed the most rapid reduction in CASE scores, while delayed combined immunotherapy was associated with relatively poorer outcomes, suggesting the importance of early combination of multiple immunotherapies and teratoma resection for anti-NMDAR encephalitis (27). Notably, we had five patients who opted for IT-MTX without second-line therapy due to severe pulmonary or urinary infections following first-line treatment. The mRS and CASE scores of these patients decreased rapidly, as did the antibody titers. This is similar to the case report, which describes rapid serological and clinical responses in patients with severe anti-NMDAR encephalitis who received combined treatment with ofatumumab and daratumumab early after failure of first-line treatment (18). But this finding needs to be confirmed with larger sample sizes and in prospective studies to rule out the effect of selection bias and to clarify the potential mechanism. Therefore, it is suggested that early intrathecal treatment can provide greater benefits to patients.

Tumors and prior infections are two well-known potential triggers (28). Approximately 58% of anti-NMDAR encephalitis cases are associated with ovarian teratomas in female patients over 18 years old (29). In our study, although one patient showed symptom relief after IT-MTX treatment, antibody titers were found to have risen again at follow-up, and a teratoma was detected at 17 months. Previous reports have described delayed teratoma development and radiologically undetectable teratomas. We hypothesize that this might reflect potential delayed teratoma growth, highlighting the necessity for long-term tumor surveillance. European guidelines recommend routine ongoing monitoring for ovarian teratomas in young female patients (≥12 years) with anti-NMDAR encephalitis, specifically pelvic MRI every 6 months to rule out teratoma recurrence within 4 years (30). Besides, one patient died after the second intrathecal injection due to severe infection, potentially related to the delayed diagnosis of a teratoma 3 months after symptom onset. Previous reports suggested that delayed initiation of treatment (31) and delayed surgical removal of teratomas may lead to poor patient outcomes. If teratoma resection is delayed, persistent antigen exposure may induce increased antibody affinity and the generation of long-lived plasma cells, rendering patients unresponsive to teratoma resection and immunotherapy (32).

Although IT-MTX was considered a generally favorable treatment for anti-NMDAR encephalitis, MTX neurotoxicity, a rare but severe complication, has been reported in other systemic immune and hematological diseases, with manifestations including encephalopathy, myelopathy, and radiculopathy (8). No similar adverse reactions have been reported in previous case reports, and in this study, none of the nine patients experienced any IT-MTX-related complications for at least 12 months of follow-up. A previous cohort study indicated that severe pneumonia, the most common complication, is a major cause of mortality in anti-NMDAR encephalitis (33). Prolonged bed rest, intubation, and immunosuppressive therapy may increase the susceptibility to infections and sepsis. Therefore, clinicians must carefully weigh the benefits and risks of immunosuppressive treatment. In our study, 9 of 10 patients developed severe pulmonary infection, and some patients even developed respiratory and circulatory failure. Fortunately, after IT-MTX treatment, eight patients had resolution of encephalitis symptoms, accompanied by a decrease in the infection index, which includes markers such as erythrocyte sedimentation rate, C-reactive protein, and procalcitonin. Furthermore, none of the nine patients developed significant complications for at least 12 months of follow-up. Therefore, IT-MTX may be a safe treatment option that can be used cautiously under close monitoring in cases of peripheral infections.

In conclusion, in our study, 10 patients diagnosed with severe anti-NMDAR encephalitis received IT-MTX therapy after an inadequate response to first-line and/or second-line treatments or due to severe infectious complications. After treatment, nine out of 10 patients had a marked response to IT-MTX with mRS scores decreasing to 0–1 and CASE scores decreasing to 0-2, along with a decrease in antibody titers. These patients remained free of complications or recurrence for at least 1 year of follow-up. Therefore, we propose that IT-MTX may be considered an escalation option following failure of second-line immunotherapy for severe anti-NMDAR encephalitis. Furthermore, early and adequate combined immunotherapy is beneficial for patients.

Our study has several limitations. First, all patients received other immunotherapies before IT-MTX administration, and the time interval between these immunotherapies and IT-MTX was variable. Therefore, the reductions in antibody titers, CASE scores, and mRS scores may not be entirely attributable to IT-MTX. Second, this is a retrospective study without a control group. Finally, the sample size was small, which reduced statistical power. More prospective studies with larger sample sizes from multiple institutions and animal experiments are needed to better illustrate the utility and mechanism of IT-MTX in severe anti-NMDAR encephalitis.

## Data Availability

The original contributions presented in the study are included in the article/supplementary material. Further inquiries can be directed to the corresponding author.
